# *Plasmodium falciparum* Infection Significantly Impairs Placental Cytokine Profile in HIV Infected Cameroonian Women

**DOI:** 10.1371/journal.pone.0008114

**Published:** 2009-12-02

**Authors:** Anfumbom Kfutwah, Jean Yves Mary, Brigitte Lemen, Robert Leke, Dominique Rousset, Françoise Barré-Sinoussi, Eric Nerrienet, Elisabeth Menu, Ahidjo Ayouba, for the ANRS 1267 study team

**Affiliations:** 1 Virology Laboratory, Centre Pasteur du Cameroun, Yaoundé, Cameroon; 2 INSERM U717, Université Paris 7, Hôpital St Louis, Paris, France; 3 Centre d'Animation Sociale et Sanitaire (CASS), Yaoundé, Cameroon; 4 Maternité principale de l'Hôpital Central, Yaoundé, Cameroon; 5 Institut Pasteur, Unité de Régulation des Infections Rétrovirales, Paris, France; Université Pierre et Marie Curie, France

## Abstract

**Background:**

Placental cytokines play crucial roles in the establishment and maintenance of pregnancy as well as protecting the foetus from infections. Previous studies have suggested the implication of infections such as *P. falciparum* and HIV in the stimulation of placental cytokines. This study assessed the impact of *P. falciparum* on placental cytokine profiles between HIV-1 positive and negative women.

**Materials and Methods:**

*P. falciparum* infection was checked in peripheral and placental blood of HIV-1 negative and positive women by the thick blood smear test. Cytokines proteins and messenger RNAs were quantified by ELISA and real time PCR, respectively. Non-parametric tests were used for statistical analyses.

**Results:**

Placental and peripheral *P. falciparum* infections were not significantly associated with HIV-1 infection (OR: 1.4; 95% confidence interval (95%CI): 0.5–4.2; p = 0.50 and OR: 0.6; 95%CI: 0.3–1.4; p = 0.26, respectively). Conversely, placental *P. falciparum* parasitemia was significantly higher in the HIV-1 positive group (p = 0.04). We observed an increase of TNF-α mRNA median levels (p = 0.02) and a trend towards a decrease of IL-10 mRNA (p = 0.07) in placenta from HIV-1 positive women compared to the HIV negative ones leading to a median TNF-α/IL-10 mRNA ratio significantly higher among HIV-1 positive than among HIV-1 negative placenta (p = 0.004; 1.5 and 0.8, respectively). Significant decrease in median secreted cytokine levels were observed in placenta from HIV-1 positive women as compared to the HIV negative however these results are somewhat indicative since it appears that differences in cytokine levels (protein or mRNA) between HIV-1 positive and negative women depend greatly on *P.falciparum* infection. Within the HIV-1 positive group, TNF-α was the only cytokine significantly associated with clinical parameters linked with HIV-1 MTCT such as premature rupture of membranes, CD4 T-cell number, plasma viral load and delay of NVP intake before delivery.

**Conclusions:**

These results show that *P. falciparum* infection profoundly modifies the placenta cytokine environment and acts as a confounding factor, masking the impact of HIV-1 in co-infected women. This interplay between the two infections might have implications in the *in utero* MTCT of HIV-1 in areas where HIV-1 and *P. falciparum* co-circulate.

## Introduction

Malaria and HIV/AIDS are two of the most common and important health problems in sub-Saharan African countries, and pregnant women are a particularly vulnerable group [1]. In pregnant women, the placenta provides a favourable environment for interactions between these two infections [2].

The placental barrier separates the mother's blood circulation from that of the fetus. The placental environment consists of several soluble factors including hormones [3], [4], maternal antibodies [5], [6] and cytokines [7]. Cytokines in particular have regulatory activities on the initiation and maintenance of pregnancy [8], [9].

In the specific case of HIV-1 infected pregnant women, cytokines interact with HIV-1 in several ways. They could either inhibit (Interferons and anti-inflammatory cytokines) [10], [11] or enhance (pro-inflammatory cytokines) HIV-1 replication [12], [13] or display both effects, depending on the target cells [14], [15]. Several authors have suggested that cytokines could be major regulators of transplacental transmission of HIV-1 [12], [16]–[20]. Moreover, granulocyte macrophage colony-stimulating factor (GM-CSF), interleukin-1 beta (IL-1β) and TNF-α have been shown to stimulate HIV-1 transcriptional modulation in placental derived trophoblastic cells [13]. Our previous studies showed that TNF-α could increase HIV-1 transcriptional modulation in infected placenta tissues [21]. Conversely, the principal characteristic of HIV-1 infection is its profound impairment of the immune system. This impairment, characterized by a generalized immune activation and a CD4 T-cell depletion has, as consequence among others, a dramatic modification of cytokine and chemokine profiles [22], [23].

Chronic immune activation is also a hallmark of the immune system of parasitized individuals in malaria endemic areas. Pregnant women are progressively less susceptible to malaria during successive pregnancies [24], [25]. This successive resistance pattern to pregnancy-associated malaria (PAM) has been related to the successive acquisition of antibodies that inhibit adhesion of *P. falciparum*–infected erythrocytes to the placental receptor, chondroitin sulphate A (CSA) [26]. This observation is consistent with evidence that these antibodies are the mediators of protective immunity to PAM [27]–[29]. Several studies have documented increased secretion of cytokines such as TNF-α, IL-1, IL-6 and IL-10 by *P. falciparum* infected placentae [25], [30]–[32]. These cytokine profile modifications during PAM have several drawbacks. For instance high levels of TNF-α have been shown to be associated with delivery of low birth weight children born to women with *P. falciparum* infected placenta [33]. Thus, malaria infection impairs normal functions of the immune system with further complications if it occurs during pregnancy.

Considering the high levels of mother-to-child transmission (MTCT) of HIV-1 in malaria endemic countries, it could be suggested that placental malaria is a risk factor associated with *in utero* MTCT of HIV-1. Malaria could achieve this role through alterations of the placental cytokine environment in HIV-1 positive women [34]. In this context, the cytokine profiles in HIV-1 infected pregnant women, as compared to a group of uninfected women could give additional information on the interplay between HIV-1 and malaria at the placenta level and in areas where both infections co-circulate.

This study was therefore initiated with the principal aim of studying the mRNA expression or secretion of placental cytokines in HIV-1 uninfected women compared to infected women who accepted to take NVP at the beginning of labour for the prevention of MTCT of HIV-1, as was practiced in this country at the time of the study, taking into account *P. falciparum* infection.

## Results

### Characteristics of the studied groups

Between the HIV-1 positive and negative pregnant women who participated in this study, there were no statistically significant differences in age, term of delivery, proportion of women with ruptured membranes more than 4 hours, birth weight of children, and the presence of infections other than HIV-1 (Hepatitis B or C Viruses, Syphilis, peripheral blood or placental *P. falciparum* infections) (**Table 1**). Nevertheless, a significant difference (p = 0.03) was observed between the two groups in the proportion of women in first pregnancy. Indeed, the present pregnancy was the first one for only 19% of HIV-1 positive women, while it represented 36% for HIV-1 negative women (**Table 1**). *P. falciparum* placental infection was not significantly associated with HIV-1 infection (odds ratio (OR): 1.4; 95% confidence interval (95%CI): 0.5–4.2; p = 0.50, **Table 1**). However parasitemia was significantly higher (p = 0.04) in the placenta of HIV-1 positive women (**Table 2**). Similarly, a trend of a higher *P. falciparum* parasitemia was also observed in the peripheral blood circulation (p = 0.08, **Table 2**), while peripheral blood *P. falciparum* infection was not significantly associated with HIV-1 infection (OR: 0.6; 95%CI: 0.3–1.4: p = 0.26, **Table 1**).

**Table 1:** General characteristics of the women recruited in the study.

Characteristics	HIV-1 Negative women	HIV-1 Positive women	*p* *
	**N = 50**	**n = 80**	
**Age at inclusion (years)**			
median (IQR)	25.2 (21.5–30.0)	26.6 (23.3–31.0)	0.10^2^
**First Pregnancy n (%)**			
Yes	18 (36)	15 (19)	0.03^1^
**Pregnancy duration (weeks)**	39.6 (38.9–40.9)	39.4 (38.3–40.6)	0.15^2^
**Birth weight (kg)**			
median (IQR)	3.3 (2.9–3.5)	3.2 (2.8–3.5)	0.58
**Ruptured membranes >4 hours**			
n (%)	7 (14)	4 (5)	0.07^2^
**Other infections at inclusion**			
**HBV** n (%)	2/50 (4)	7/78 (9)	0.28
**HCV** n (%)	2/50 (4)	1/80 (1)	0.31
**Syphilis** n (%)	1/21 (5)	6/65 (9)	0.51
**Peripheral blood ** ***P.falciparum*** **, n (%)**	14/50 (28)	15/77 (19)	0.26
**Placental ** ***P.falciparum*** **, n (%)**	8/45 (18)	9/38 (24)	0.50

* Median values and Interquartile ranges (IQR) are presented. 1 = Significant difference (p<0.05); 2 = tendencies (0.15>p>0.05); ND = not determined.

**Table 2:** *P. falciparum* parasitemia among HIV-1 negative and positive pregnant women in Yaoundé.

	HIV Negative	HIV positive	p*
**Peripheral Blood parasitemia**	**n = 14**	**n = 14** 1	**0.08**
≤1/1000	13	8	
2/1000–5/1000	1	3	
≥6/1000	0	3	
**Placental Parasitemia**	**8**	**9**	**0.04**
≤1/1000	8	4	
2/1000–5/1000	0	1	
≥6/1000	0	4	

p*: Chi Square Test.

1 Peripheral blood parasitemia was not determined in 1 HIV-1 positive woman with peripheral blood *P-falciparum* infection.

Significantly lower median haemoglobin (p<0.001) and neutrophils (p = 0.002) levels were observed in HIV-1 positive compared to HIV-1 negative women (Supplementary **Figure S1**). HIV-1 specific characteristics indicate a median viral load of 4.0 log_10_ (inter-quartile range (IQR) of 3.1–4.5 log_10_), a median CD4+ T-cell count at delivery of 364 cells/mm^3^ (IQR = 245–504 cells/mm^3^). The median time of NVP intake before delivery was 5 hours (IQR = 4–8 hours). Sixty-four of the 80 children delivered to HIV-1 positive women were tested for HIV-1 infection 6 to 8 weeks after birth. Seven (11%) of the 64 children were HIV infected and presented high viral load, between 5 and 5.7 log_10_ during the first 6 to 8 weeks of life. All of the 7 infected children were of the female gender.

### Comparison of placental cytokine profiles between HIV-1 positive and negative women

**Figure 1:**
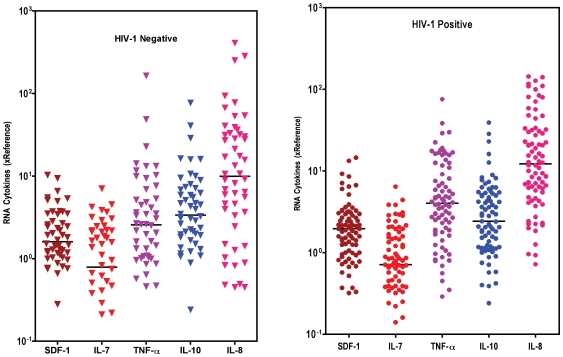
Levels of cytokine mRNA expressed in the placenta of HIV-1 negative (left panel) and positive (right panel) women. Cytokine mRNA were extracted from Cryo-preserved placenta fragments of HIV-1 negative and positive women. Reverse transcribed cytokine mRNA were quantified by Real Time PCR. Results were normalized to references values of a HIV-1 negative placenta (Ref). Each dot represents one individual placenta and the horizontal bar the median value. A total number of 50 placentas for HIV-1 negative and 80 for HIV-1 positive women were tested for each cytokine. Comparison between the 2 groups was performed through Mann-Whitney test.

The levels of placental cytokine mRNA expression of SDF-1, IL-7, IL-10, TNF-α, and IL-8 were compared between HIV-1 positive and negative women. As shown in **Figure 1**, variable levels of each cytokine were observed within each group (HIV-1 positive or negative). The levels of IL-8 were high in both groups, while expression levels of IL-7 were the lowest. The median levels of RNA expression for any given cytokine, except TNF-α and IL-10, were similar in HIV-1 negative and positive women. For TNF-α, there was a significant increase (p = 0.02) and a trend of an IL10 decrease (p = 0.07) in HIV positive women as compared to HIV negative women, leading to a significant increase of the ratio TNF-α/IL10 (p = 0.004). The median TNF-α/IL-10 ratio among HIV-1 positive women (median: 1.5, inter-quartile range: 0.7–4.1) was significantly higher than among HIV-1 negative women (median: 0.8; inter-quartile range: 0.4–1.8).

**Figure 2:**
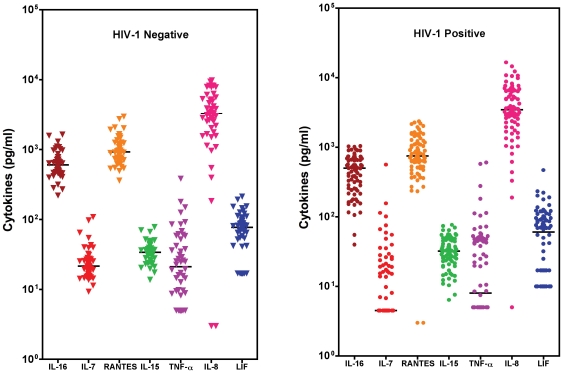
Cytokine protein secretion in the culture supernatant after a 24 hour explant culture of placenta of HIV-1 negative (left panel) and positive (right panel) women. Placental fragments were cultured for 24 hours after which culture supernatants were harvested. Cytokines were quantified in the supernatants using commercially available ELISA kits. Each dot represents one individual placenta and the horizontal bar, the median value. A total number of 50 placentas for HIV-1 negative and 80 for HIV-1 positive women were tested for each cytokine. For IL7, TNF-α, IL-8 and LIF, the lowest values correspond to the assay detection limit. Comparisons between the 2 groups were performed through Mann-Whitney test.

After a 24-hour culture of fresh placental fragments, cytokine secretion levels were measured in the culture supernatants (**Figure 2**). The following cytokines were analysed and compared between HIV-1 positive and negative women: IL-16, IL-7, RANTES, IL-15, TNF-α, IL-10, IL-8, and LIF. In both groups, high and variable values were observed for IL-16, RANTES and IL-8, while low values were observed for IL-7, IL-15 and LIF, low to undetectable levels were obtained for IL-10 (data not shown). In 0%, 94%, 22%, 4% and 22% of HIV-1 negative placentas and in 56%, 100%, 49%, 1% and 41% of HIV-1 positive placentas, secretion levels of IL-7, IL-10, TNF-α, IL8 and LIF, respectively, were not detectable. TNF-α to IL10 ratio could not be calculated at the protein level because IL-10 protein in the supernatants was below the detection level (5pg/ml) for all the 80 placentae of HIV-1 positive women and for 47 out of 50 placentae of HIV-1 negative women. IL7, TNF-α, LIF, IL16 and RANTES median levels were decreased in HIV positive women as compared to HIV negative women (p<0.001, p = 0.07, p = 0.03, p = 0.004 and p = 0.04, respectively).

The placental cytokine expression and secretion levels (mRNA and protein) between the 7 transmitting and the 57 non-transmitting HIV-1 positive mothers (data not presented) were not significantly different.

### The pro-inflammatory cytokine TNF-α is strongly associated with clinical parameters known to be related to MTCT of HIV-1

When analysing the association between placental cytokine levels and the 9 clinical parameters investigated (the 6 parameters described in table 1, plus for HIV-1 positive women, viral load, CD4 T-cell number and delay between nevirapine uptake and delivery), the pro-inflammatory cytokine TNF-α plays a particular role with an association in 6 out of the 9 parameters, whereas one or no association was observed for the other cytokines (**Table 3**). Moreover, this association was observed with all the 4 clinical parameters known to be related to MTCT of HIV-1. The proportion of placenta with undetectable TNF-α protein level was significantly associated with plasma viral load over 4.5 in log_10_/ml (73% of values lower that the detection level (10pg/ml) versus 40%, p = 0.008), with high delay between nevirapine uptake and delivery (71% of values lower than the detection limit, versus 33%, p = 0.007), with CD4 T-cell number over 250/mm^3^ (median level lower than the detection limit versus 49 pg/ml, p = 0.04). In addition, high TNF-α expression level was significantly associated with CD4 T-cell number over 250/mm^3^ (median level of 4.8×reference versus 2.8, p = 0.02). These associations between TNF-α protein secretion or expression levels and CD4 T-cell numbers were confirmed through significant Spearman correlation coefficient (−0.29, p = 0.01 and +0.27, p = 0.02, respectively). When analysing the association between placental cytokine levels and the 6 parameters investigated in HIV-1 negative women, one or no association was observed for all cytokines.

**Table 3:** Association between clinical parameters and placental cytokines in HIV-1 positive women.

	Ruptured membranes (≥4h)	CD4	Viral load	NVP administration before delivery	Infection other than HIV	Parity	Number of previous Pregnancies	Pregnancy duration	Birth weight
	Correlation (p-value)	Correlation (p-value)	Correlation (p-value)	Correlation (p-value)	Correlation (p-value)	Correlation (p-value)	Correlation (p-value)	Correlation (p-value)	Correlation (p-value)
TNF-α transcript	**positive (0.02)**	**positive (0.02)**	(NS)	(NS)	(NS)	(NS)	(NS)	(NS)	(NS)
TNF-α protein	**negative (0.05)**	**negative (0.004)**	**negative (0.01**)	(NS)	(NS)	**positive (0.04)**	**positive (0.03)**	(NS)	(NS)
TNF-α undetectable	(NS)	**positive (0.04)**	**positive (0.008)**	**positive (0.007)**	(NS)	**negative (0.005)**	**negative (0.004)**	(NS)	(NS)
IL-8 protein	(NS)	(NS)	(NS)	(NS)	(NS)	(NS)	(NS)	**negative (0.002)**	(NS)
IL-16 protein	(NS)	(NS)	(NS)	(NS)	**positive (0.03)**	(NS)	(NS)	(NS)	(NS)
LIF protein	(NS)	(NS)	**negative (0.004)**	(NS)	(NS)	(NS)	(NS)	(NS)	(NS)
LIF undetectable	(NS)	(NS)	(0.003)	(NS)	(NS)	(NS)	(NS)	(NS)	(NS)
IL-15 protein	(NS)	(NS)	(NS)	**negative (0.05)**	(NS)	(NS)	(NS)	(NS)	(NS)
IL-7 protein	(NS)	(NS)	(NS)	(NS)	(NS)	(NS)	(NS)	(NS)	**negative (0.03)**
RANTES protein	(NS)	(NS)	**positive** (0.05)	(NS)	(NS)	(NS)	(NS)	(NS)	(NS)

NS: not significant. See Methods section for P-value calculation.

### Placental cytokine profiles differ significantly between HIV-1 positive and negative women, when taking into account *Plasmodium falciparum* infection

The placental cytokine profiles were next analysed taking into consideration the *P. falciparum* infection status of the women both in the placenta and in the peripheral blood.

Messenger RNA or protein cytokine levels were compared between HIV-1 positive and negative women in the presence or absence of infection by *P. falciparum* in the peripheral blood (**Tables 4** **and** **5**) or in the placenta (**Tables 6** **and** **7**). When peripheral blood *P. falciparum* was negative, numerous significant differences and trends were observed in placental cytokine levels between HIV-1 negative and positive women (**RNA-** TNF-α, IL-10 and TNF-α/IL-10, table 4; **Proteins-** IL-16, IL-7, undetectable IL-7, TNF-α, undetectable TNF-α, LIF, undetectable LIF, and RANTES, table 5). Hence, there were significant lower levels of TNF-α mRNA and TNF-α/IL-10 mRNA ratio in peripheral blood *P. falciparum* negative women HIV-1 uninfected compared to peripheral blood *P. falciparum* negative women HIV-1 infected (Table 4). On the other hand in peripheral blood *P. falciparum* positive women, fewer significant differences were observed in placental cytokine levels between HIV-1 positive and negative women (**RNA-** IL-8, table 4a; **Proteins-** IL-7, undetectable IL-7, table 5).

**Table 4:** Difference in placental cytokine expression levels (RNA) between HIV-1 negative and positive women in relation to peripheral blood *Plasmodium falciparum* status.

Cytokines	Pb *P.falciparum* negative			Pb *P.falciparum* positive		
	HIV-1 Negative (n = 36)	HIV-1 Positive (n = 62)	p	HIV-1 Negative (n = 14)	HIV-1 Positive (n = 15)	p*
**RNA (x ref)**						
SDF-1	1.5 (1.2–3.3)	2.0 (1.0–3.0)^*^	0.97	1.8 (0.8–3.5)	1.7 (1.0–3.5)	0.54
IL-7	0.7 (0.0–2.1)	0.7 (0.3–1.7)	0.88	1.4 (0.1–2.5)	0.7 (0.5–1.5)	0.74
TNF-α	**1.8 (0.9–6.5)**	**4.2 (2.3–12.5)**	**0.009**	3.2 (2.0–5.8)	3.2 (1.1–5.7)	0.79
IL-10	**3.1 (1.8–7.3)**	**2.1 (1.1–5.0)**	**0.07^2^**	4.8 (1.3–7.4)	3.6 (1.2–5.1)	0.28
IL-8	16.4 (2.7–40.1)	11.4 (5.3–31.0)	0.84	**8.0 (3.3–17.0)**	**12.6 (6.7–32.2)**	**0.12^2^**
TNF-α/IL-10	**0.8 (0.3–1.9)**	**2.0 (0.7–5.1)**	**0.001^1^**	0.9 (0.4–2.0)	1.0 (0.4–1.6)	0.73

The mRNA expression levels are referenced to a term placental tissue (reference calibrator: cal). The amount of target gene in the sample and in the calibrator was normalised to the endogenous reference (18S RNA). The values are indicated as median and inter-quartile range. p*: Mann-Whitney Test; Pb = peripheral blood; ^1^ = significant difference (P<0.05); ^2^ = trend (0.05<p<0.15).

**Table 5:** Difference in placental cytokine secretion levels (Proteins) between HIV-1 negative and positive women in relation to peripheral blood *Plasmodium falciparum* status.

	Pb *P.falciparum* negative			Pb *P.falciparum* positive		
	HIV-1 Negative (n = 36)	HIV-1 Positive (n = 62)	p*	HIV-1 Negative (n = 14)	HIV-1 Positive (n = 15)	p*
**Protein (pg/ml)**						
IL-16	**569 (480–674)**	**417 (227–672)**	**0.007^1^**	685 (449–991)	596 (441–764)^**^	0.41
IL-7	**21 (16–26)**	**<9 (<9–24)**	**<0.001^1^**	**25 (19–34)**	**<9 (<9–14)**	**<0.001^1^**
IL-7 Undetectable	**0%**	**53%**	**<0.001^1^**	**0%**	**67%**	**<0.001^1^**
TNF-α	**18 (<10–54)**	**<10 (<10–45)**	**0.05^1^**	25 (<10–62)	20 (<10–48)	0.72
TNF-α Undetectable	**19%**	**53%**	**0.001^1^**	29%	33%	0.78
LIF	**81 (42–104)**	**42 (<19–96)**	**0.01^1^**	68 (35–97)	83 (<19–112)	0.83
LIF Undetectable	**22%**	**45%**	**0.02^1^**	21%	33%	0.47
IL-8	3355 (1841–5245)	3460 (2288–6760)	0.63	3121 (1349–5335)	2935 (2114–5368)	0.66
IL-8 Undetectable	0%	2%	0.44	14%	0%	0.22
IL-15	34 (25–44)	31 (23–46)	0.46	34 (32–39)	32 (21–38)	0.28
RANTES	**981 (747–1541)**	**795 (529–1251)**	**0.03^1^**	770 (657–1201)	713 (630–1057)	0.86

The values are indicated as median and inter-quartile range. p*: Mann-Whitney Test; Pb = peripheral blood; ^1^ = significant difference (P<0.05); ^2^ = trend (0.05<p<0.15); **n = 14.

**Table 6:** Difference in Placental cytokine expression levels (RNA) between HIV-1 negative and positive women in relation to Placental *Plasmodium falciparum* status.

Cytokines	Pl *P.falciparum* Negative			Pl *P.falciparum* Positive		
	HIV-1 Negative (n = 37)	HIV-1 Positive (n = 29)	p*	HIV-1 Negative (n = 8)	HIV-1 Positive (n = 9)	p*
**RNA (x ref)**						
SDF-1	1.5 (1.0–3.6)	2.1 (0.8–3.0)^*^	0.87	1.6 (1.2–2.3)	1.3 (1.0–2.0)	0.70
IL-7	**0.8 (0.0–2.4)**	**0.4 (0.1–0.8)**	**0.10^2^**	0.8 (0.0–2.1)	1.3 (0.7–1.7)	0.70
TNF-α	**2.2 (0.9–5.0)**	**4.5 (1.2–10.8)**	**0.12^2^**	3.9 (1.1–9.4)	3.9 (2.7–16.6)	0.92
IL-10	**3.3 (1.5–7.6)**	**1.9 (1.1–5.4)**	**0.08^2^**	5.0 (3.4–7.0)	3.6 (1.4–5.6)	0.34
IL-8	15.8 (1.5–37.8)	10.1 (4.5–35.2)	0.87	8.0 (5.8–12.7)	12.6 (6.6–25.6)	0.34
TNF-α/IL-10	**0.8 (0.3–1.8)**	**1.6 (0.5–4.8)**	**0.03^1^**	0.8 (0.3–1.2)	1.1 (0.5–5.7)	0.44

The mRNA expression levels are referenced to a term placental tissue (reference calibrator: cal). The amount of target gene in the sample and in the calibrator was normalised to the endogenous reference (18S RNA). The values are indicated as median and inter-quartile range. p*: Mann-Whitney Test; Pl = placental; 1 = significant difference (P<0.05); 2 = trend (0.05<p<0.15); * n = 28.

**Table 7:** Difference in Placental cytokine secretions levels (Protein) between HIV-1 negative and positive women in relation to Placental *Plasmodium falciparum* status.

Cytokines	Pl *P.falciparum* Negative			Pl *P.falciparum* Positive		
	HIV-1 Negative (n = 37)	HIV-1 Positive (n = 29)	p*	HIV-1 Negative (n = 8)	HIV-1 Positive (n = 9)	p*
**Protein (pg/ml)**						
IL-16	557 (473–695)	559 (283–819)^**^	0.79	**926 (632–1087)**	**666 (532–706)**	**0.08^2^**
IL-7	**21.0 (16.5–26.3)**	**<9 (<9–22.2)**	**0.001^1^**	**29.2 (25.3–51.0)**	**<9 (<9–23.8)**	**0.02^1^**
IL-7 undetectable	**0%**	**55%**	**<0.001^1^**	**0%**	**56%**	**0.01^1^**
TNF-α	25.3 (13.6–58.7)	29.0 (<10–52.0)	0.68	16.1 (6.1–125.7)	24.9 (<10–49.6)	0.77
TNF-α undetectable	**14%**	**37%**	**0.04^1^**	25%	33%	0.71
LIF	81 (47–112)	76 (<19–107)	0.61	70 (45–87)	94 (44–115)	0.16
LIF undetectable	22%	30%	0.39	13%	22%	0.60
IL-8	3523 (1620–5273)	2935 (1653–4784)	0.41	2835 (1368–3370)	3916 (2686–6836)	0.15
IL-8 Undetectable				12%	0%	0.27
IL-15	34 (28–42)	33 (26–53)	0.86	33 (23–42)	36 (26–47)	0.29
RANTES	**953 (737–1321)**	**790 (511–1252)**	**0.09^2^**	758 (570–1126)	703 (620–1031)	0.77

The mRNA expression levels are referenced to a term placental tissue (reference calibrator: cal). The amount of target gene in the sample and in the calibrator was normalised to the endogenous reference (18S RNA). The values are indicated as median and inter-quartile range. p*: Mann-Whitney Test; Pl = placental; 1 = significant difference (P<0.05); 2 = trend (0.05<p<0.15); **n = 27.

A similar observation was made when placental *P. falciparum* was negative (Tables 6 and 7), several significant differences and trends were observed in placental cytokine levels between HIV-1 positive and negative women (**RNA-** IL-7, TNF-α, IL-10 and TNF-α/IL-10, table 6; **Proteins-** IL-7, undetectable IL-7, undetectable TNF-α, RANTES table 7). When placentas were *P. falciparum* positive, fewer placental cytokine levels were significantly different between HIV-1 positive and negative pregnant women (**RNA-** IL-7, table 6; **Proteins-** IL-16, IL-7, undetectable IL-7, table 7).

There were significantly lower levels of TNF-α mRNA and TNF-α/IL-10 mRNA ratio in placenta *P.falciparum* negative women HIV-1 uninfected compared to placenta *P.falciparum* negative women HIV-1 infected (Table 6).

It should be noted the lost of significant difference in the TNF-α/IL-10 mRNA ratio between HIV-1 negative and positive women in the presence of *P. falciparum* infection in both peripheral blood and placenta (Tables 4 and 6).

Thus, placental cytokine profiles are profoundly modified by *P. falciparum* infection, regardless of whether it is placental or systemic malaria.

## Discussion

The aim of this study was to assess the human placental cytokine profiles attributable to HIV-1 infection in an African setting where malaria and HIV infections are both endemic. Our analysis of raw data revealed significant correlations between cytokines and HIV associated clinical parameters with significant decrease of IL-7, TNF-α, LIF, IL-16 and RANTES proteins median levels in HIV positive women. These changes were however only meaningful if malaria infection was considered as differences between the two groups were dependant on the proportion of malaria positive samples in each group. After stratification for the presence of malaria in peripheral blood or within the placenta, highly significant differences between the two groups were observed for both expressed and secreted cytokines.

In this study we observed a similar distribution of clinical parameters (age, duration of pregnancy, parity, body weight of babies at delivery and the prevalence of other infections such as *P. falciparum*, HBV, HCV and *T. pallidum*) in HIV-1 negative and positive women. This finding is justified by the fact that HIV-1 positive and negative women were drawn from the same environment and that HIV positive women included in this study were not severely immuno-depressed as exemplified by an average CD4 T-cell count of about 364 cells/mm^3^. The significantly lower levels of haemoglobin observed among HIV-1 positive women (supplementary figure S1) is in agreement with earlier studies [35], [36], as well as the lower neutrophil counts which have been proposed by some authors as due to a direct infection of a neutrophil sub population expressing CD4 and CXCR4 surface markers [37]. Another reason could be the fact that neutrophils in HIV positive women are depleted by Fas (CD95) mediated apoptosis [38]

Several studies reported significant differences in placental cytokine levels between HIV-1 negative and positive women [12], [39], [40]. In the present work, of the studied cytokines, TNF-α only was significantly increased in HIV positive women at RNA level. All the remaining cytokines were of comparable levels between the two groups, contrasting with these earlier findings. At the protein level, however, significant differences were observed between HIV negative and HIV positive placenta cytokines, with a systematic decrease in the group of HIV positive women. We hypothesised that *P. falciparum* could masks some differences in placental cytokine levels between HIV-1 negative and positive women since *P. falciparum* have an impact on the immune system, especially of pregnant women and is endemic to the study site. In this study, we observed similar *P. falciparum* infection rates in both groups. Conversely, there was a significantly higher parasite density (parasitemia) among the HIV-1 positive women both in the placenta as well as in the peripheral blood circulation. This result indicates that both groups are equally exposed to *P. falciparum* infection, but that HIV-1 positive women less efficiently limit *P. falciparum* propagation. *P. falciparum* is known to sequestrate in the placenta leading to the accumulation of parasitized erythrocytes in the intervillous space, infiltration of inflammatory cells and release of some placental cytokines [25], [41]. The release of these cytokines as a result of *P. falciparum* sequestration in the placenta significantly modifies the placental cytokine environment [31], [32], [42]. Analysis of our data, considering the *P. falciparum* status, showed that there were significant differences in the placental cytokine levels of HIV-1 negative and positive women (Table 6 and 7). In the absence of *P. falciparum* infection, significant differences in the levels of several placental cytokines were observed between HIV-1 negative and positive women. However in women who were *P. falciparum* positive, fewer cytokines were significantly different between HIV-1 negative and positive women. Hence, the differences observed without stratification with malaria between HIV-1 positive and negative groups was dependant on the proportion of malaria positive samples in each of the two groups. Indeed, since differences in cytokine mRNA expression or secretion levels between HIV positive and HIV negative women are different in malaria infected and malaria non-infected women, as we clearly observed. Thus, by altering the normal placental cytokine environment, *P. falciparum* infection modifies or hides the impact of HIV-1 on the very same environment.

In this study, *P. falciparum* is playing a confounding role in placental cytokine analyses between HIV-1 negative and positive women and should be taken into consideration when comparing placental cytokines in malarial endemic zones like Cameroon. Modification of the placental cytokine environment could have varying consequences in MTCT of HIV-1. The precise mechanism by which malaria and HIV infections interact to influence MTCT of the virus still remains to be uncovered. However, several studies have pointed out the predominant role of pro-inflammatory cytokines, especially TNF-α, on this interference. Several groups in Cameroon have shown that TNF-α is secreted in the placenta infected by *P. falciparum* [31], [32].

In our study, no difference in placental cytokine profiles could be evidenced between transmitting and not transmitting mothers, but this is likely to be due to the few number of HIV-1 infected children. When looking for associations between placental cytokine levels and clinical parameters, the observation of the association between a cytokine level and only one clinical parameter was considered as chance related due to the numerous tests of association performed. Not surprisingly, the only placental cytokine that was associated to several clinical parameters was TNF-α. Especially, TNF-α level was associated with all the four clinical parameters studied here known to be related to MTCT of HIV-1. However, the negative sign of association between TNF-α (protein) with CD4, plasma viral load and rupture of membrane more than 4 hours is surprising. A plausible explanation could be again the confounding effect of *P. falciparum*. Other groups have shown this cytokine in the placenta to be associated with low birth weight [30], [33] another risk factor for MTCT of HIV-1. Several authors have reported that TNF-α stimulates HIV-1 replication in infected cell lines by an LTR-driven manner [13], [43]. In particular our group showed that TNF-α stimulates HIV-1 replication in human term placentas obtained after elective caesarean section [21]. Our data on the associations of these clinical parameters with TNF-α essentially due to pregnancy associated malaria are thus in line with these previous observations. It is noteworthy that, at the mRNA level, the unique statistically significant difference in placenta cytokines of HIV-1 infected women and uninfected controls observed in the present study was on the ratio TNF-α to IL-10 levels. Indeed, TNFα and IL-10 are two prototypic pro-and anti-inflammatory cytokines, respectively, whose roles in HIV-1 replication and during malaria infection are well described [13], [14], [32], [41], [44], [45], This difference is not significant anymore in the presence of *P. falciparum* infection. This observation underlines the importance of the notion of the pro- and anti- inflammatory balance, especially for these two major cytokines, in the context of pregnancy and infection with pathogens [13], [46], [47].

Considering the high prevalence of *P. falciparum* among pregnant women observed in this study and others [48], we could therefore expect an HIV-1/*P.falciparum* interaction at the level of the placenta and this could probably explain the high levels of *in utero* MTCT of HIV-1 observed during malaria peak periods in the rainy season [49]. The *P. falciparum*/HIV-1 interactions in the placenta are also an important morbidity factor [50].

This study was part of a multicentric study comprising two other sites, France where combined therapies were administered to pregnant women for PMTC of HIV-1 and Thailand where bi-therapy was administered. Both sites had very little or insignificant infections by *P.falciparum*. Considering that only a single dose of NVP was administered to the women in Cameroon just before delivery, it could be suggested, on the basis of the short exposure time to this single dose monotherapy, that there was little or no effect on the placental cytokine environment due the drug. On the other hand, our collaborators from the French and Thai sites showed evidence of impact of ARV on placental cytokines [40], [51].

The take home message of our results here is that *P. falciparum* infection plays a significant role in altering the placental subtle equilibrium of cytokine profiles and is a confounding factor for the comparison between HIV negative and positive women. This underlines the complex impact and interaction of both pathogens at the level of the placenta. This should be taken into consideration in further studies aiming to evaluate the impact of *P.falciparum* on HIV-1 MTCT.

## Materials and Methods

### Ethical consideration

The study was reviewed and approved by the Cameroon national ethical committee and by the Cameroon Health authorities. All women included in the study gave their written informed consent.

### Study site

The present study was carried out in the framework of a public health program for the prevention of HIV-1 mother-to-child transmission, in Yaoundé, Cameroon [52]. The study sites included the maternity of the Central Hospital, the Chantal Biya Mother and Child Healthcare centre (FCB) and the CASS of Nkolndongo in Yaoundé. All the biological analyses were carried out in the Centre Pasteur du Cameroun (CPC).

### Subjects

Pregnant women were recruited within these sites between June 2001 and October 2004. The HIV sero-status of the women was determined by applying the national diagnostic algorithm of two rapid tests. Indeterminate cases were confirmed in the CPC using a previously described algorithm [52]. During that period, 80 HIV-1 positive women were included between the beginning of their second trimester of gestation and delivery. Only women infected by HIV-1 belonging to the Major group (group M) and not under ARV therapy for their disease were included. In addition, they should have agreed to take a tablet of nevirapine at the beginning of labour and also a single dose was given to the baby within the first 72 hours of life as practiced in Cameroon at the time of the study. Inclusion of HIV-1 negative women was initiated in September 2002 and 50 of them were included, mostly at delivery. Whatever HIV-1 status, women recruited before delivery were regularly followed-up until they delivered. Women were excluded from the study if they were not resident in Yaoundé or if they did not give a written informed consent. Women with multiple pregnancies or those who delivered through caesarean sections were also excluded.

### Biological samples

At inclusion, 2×5ml of maternal venous blood was collected in EDTA-treated tubes for whole blood count, malaria parasite, Hepatitis B- and C-viruses, and Syphilis diagnoses. In addition, CD4 counts and viral load quantification were performed for HIV-1 positive pregnant women on the same samples. Placentas were collected and processed within 3 hours following delivery. From the placentas, different types of samples were prepared. Firstly, thick and thin blood films from the maternal side of the placentas were prepared to look for malaria parasites. Secondly, after extensive washings with phosphate buffered saline (PBS), chorionic villi explants (3g/20 ml of culture medium) were cultured for 24 hours at 37°C in humidified atmosphere containing 5% CO_2_. Thirdly, after extensive washings, chorionic villi fragments were stored in RNAlater (Qiagen) for subsequent RNA extraction and cytokine RNA quantification by real time PCR (ABIPrism 7000). Placenta preparation for malaria as described above could only start in October 2002 and was thus performed on placenta from 45 HIV-1 negative women and 38 HIV-1 positive women.

### Complete blood count

An automated counter (ABX Pentra 120) was used to measure complete blood cell count, the quantity and percentages of each type of blood cells was tallied, including neutrophils. Haemoglobin was also quantified.

### Malaria parasite screening

Thin and thick blood and placenta smears were prepared, stained with Giemsa and examined microscopically for malaria parasites. Parasite load was expressed as the number of asexual forms of *P. falciparum* per 1000 red blood cells.

### Serologies (HIV, HBV, HCV and Syphilis)

Commercial Enzyme Immuno Assay (EIA) kits were used for plasma analysis. HIV screening was performed using an algorithm of rapid tests as approved by the National Committee for the Fight against HIV in Cameroon. Positive cases were confirmed in the laboratory of virology of the Centre Pasteur du Cameroun as previously reported [52]. HBV (MONOLISA Ag HBs PLUS BIO-RAD, France) and HCV (MONOLISA HCV PLUS version 2, BIO-RAD, France) were tested following manufacturer's instructions. A combination of two tests was used for syphilis diagnosis, namely VDRL (BioMérieux) followed by TPHA (BioRad). CD4 cell counts were performed using the flow cytometry technique (Becton Dickinson FACSCount™ System). Direct quantification of viral particles was done using the branched-DNA technique (Quantiplex® HIV-RNA).

### Secreted cytokine quantification by ELISA

After extensive washing to remove residual maternal blood, three grams of placental fragments were cultured in 20ml of culture medium made up of RPMI 1640 (Gibco BRL life technologies) supplemented with 10% fetal bovine serum, 1% L-glutamine (Gibco BRL life technologies), and 1% penicillin/streptomycin (Gibco BRL life technologies). After 24 hours of culture at 37°C and 5% CO_2_, culture supernatants were harvested, centrifuged at 3 000 rpm for 30 minutes and several aliquots were stored at −80°C for cytokine quantification by ELISA. Several cytokines including pro-inflammatory, Th1/Th2 type cytokines, and chemokines, were assayed using quantitative commercial sandwich enzyme immunoassay kits. The following cytokines were analyzed: TNF-α (Cell Com, Beckman Coulter Company; sensitivity 10pg/ml), IL-8 (Quantikine, R&D systems; sensitivity 10pg/ml), RANTES (Quantikine, R&D systems; sensitivity 8pg/ml), IL-15 (Quantikine, R&D systems; sensitivity 2pg/ml), LIF (Screening done by an 〈〈in house〉〉 test of the laboratory of Immunology, University of Bordeaux 2; sensitivity 19 pg/ml) [53], IL-7 (BioSource International; sensitivity 9pg/ml), IL-16 (BioSource International; sensitivity 5pg/ml) and IL-10 (Cell Com, Beckman Coulter Company; sensitivity 5 pg/ml).

### Cytokine RNA quantification by real time PCR

RNA later cryo-conserved and washed placental fragments were brought to room temperature, RNAs extracted using the RNeasy Mini kit (Qiagen) and stored in RNA guard (Amersham Pharmacia Biotech Inc). Reverse transcriptase PCR was performed using the RT reaction kit (Applied Biosystems, Roche) consisting of the following: 10× RT Buffer, 5.5 mM MgCl_2_, 500 µM/dNTP, 2.5µM Random Hexamer, 0.4 unit/µl RNase inhibitor, 1.25unit/µl Multiscribe RT, RNA sample (10pg–2µg) and the thermal cycler was programmed with an incubation period of 10 minutes at 25°C, reverse transcription at 48°C for 30 minutes and reverse transcriptase inactivation at 95°C for 5 minutes. The ensuing cDNAs were quantified using ABIPrism 7000 (Applied Biosystems).

For mRNA quantification, pre-developed reagents from Applied Biosystems were used for IL-7, IL-8 and TNF-α while “in house” developed primers and probes were used for SDF-1 and IL-10; 20× PreDev Target Mixed Reagents (PE Applied Biosystems) were used together with 20× human 18S rRNA Pre-developed Taqman assay (internal controls), 5µl of cDNA and the total volume adjusted to 25µl using RNase/DNase-free Water (Bio 101 Systems). The specific oligonucleotide primer sets and *Taq*man® probes used to amplify SDF-1 and IL-10 have been described previously [54]. SDF-1 and IL-10 primers and probes were used together with 20× human 18S rRNA Pre-developed Taqman assay, 5µl of cDNA and volume adjusted to 25µl using RNase/DNase-free Water (Bio 101 systems). The thermal cycler was programmed with the following conditions: 50°C for 2 minutes, 95°C for 10 minutes and then the amplifications with an initial denaturation step at 95°C for 15 seconds followed by annealing/extension at 60°C for 1 min for a total of 40 cycles. All samples were treated in triplicates. Results are presented in comparison to a reference HIV-1 negative placenta.

### Statistical analysis

The profiles of secreted cytokines (ELISA) among HIV-1 positive and negative women who were *P. falciparum* positive or negative in the peripheral blood circulation or in the placenta is described through median and inter-quartile range (25^th^ and 75^th^ percentiles) and/or by the proportion of samples with undetectable level in each group. The placental cytokine (RNA) profiles are described in the same way within each group through median and inter-quartile range. The comparison of these distributions between the groups was performed through non-parametric tests, Kruskal-Wallis test in case the number of groups compared is more than two or the Mann-Whitney test in case two groups are compared. As regards proportion of undetectable values, comparisons were realised with the chi square test or the Fisher's exact test when necessary. Association in HIV-1 positive women between the different cytokine protein secretions or mRNA expressions and clinical parameters known to be related with MTCT of HIV-1 was tested through Spearman correlation test for quantitative clinical parameters and Mann-Whitney or Kruskal-Wallis test for qualitative ones. Significant differences or associations were defined by a p<0.05 whereas trends were observed for p values between 0.05 and 0.15.

## Supporting Information

Figure S1 Box-plot of hemoglobin (bottom panel) and neutrophil (upper panel) distributions in the blood of HIV-1 negative and positive pregnant women. Hemoglobin and neutrophil levels were quantified in blood samples of pregnant women by an automated counter. Distributions are represented per group as median, inter-quartile range, 95% confidence interval and values outside this interval. Comparisons between the 2 HIV-1 groups were performed through Mann Whitney test.
(1.16 MB TIF)

## References

[1] Gavin L, Galavotti C, Dube H, McNaghten AD, Murwirwa M (2006). Factors associated with HIV infection in adolescent females in Zimbabwe.. J Adolesc Health.

[2] Tkachuk AN, Moormann AM, Poore JA, Rochford RA, Chensue SW (2001). Malaria enhances expression of CC chemokine receptor 5 on placental macrophages.. J Infect Dis.

[3] Bourinbaiar AS, Nagorny R (1992). Effect of human chorionic gonadotropin (hCG) on reverse transcriptase activity in HIV-1 infected lymphocytes and monocytes.. FEMS Microbiol Lett.

[4] Lacroix MC, Guibourdenche J, Fournier T, Laurendeau I, Igout A (2005). Stimulation of human trophoblast invasion by placental growth hormone.. Endocrinology.

[5] Story CM, Mikulska JE, Simister NE (1994). A major histocompatibility complex class I-like Fc receptor cloned from human placenta: possible role in transfer of immunoglobulin G from mother to fetus.. J Exp Med.

[6] Barin F, Jourdain G, Brunet S, Ngo-Giang-Huong N, Weerawatgoompa S (2006). Revisiting the Role of Neutralizing Antibodies in Mother-to-Child Transmission of HIV-1.. J Infect Dis.

[7] Chaouat G, Cayol V, Mairovitz V, Dubanchet S (1999). Localization of the Th2 cytokines IL-3, IL-4, IL-10 at the fetomaternal interface during human and murine pregnancy and lack of requirement for Fas/Fas ligand interaction for a successful allogeneic pregnancy.. Am J Reprod Immunol.

[8] Saito S (2000). Cytokine network at the feto-maternal interface.. J Reprod Immunol.

[9] Fox H, WBSC Ltd (ed.) (1997). Physiology of the placenta.. Pathology of the placenta, 2nd ed.

[10] Soumelis V, Scott I, Liu YJ, Levy J (2002). Natural type 1 interferon producing cells in HIV infection.. Hum Immunol.

[11] Pedroza-Martins L, Boscardin WJ, Anisman-Posner DJ, Redsar BN, Bryson YJ (2006). Interferon-gamma decreases replication of primary R5 HIV-1 isolates in thymocytes.. Aids.

[12] Lee BN, Ordonez N, Popek EJ, Lu JG, Helfgott A (1997). Inflammatory cytokine expression is correlated with the level of human immunodeficiency virus (HIV) transcripts in HIV-infected placental trophoblastic cells.. J Virol.

[13] Zachar V, Fink T, Koppelhus U, Ebbesen P (2002). Role of Placental Cytokines in Transcriptional Modulation of HIV Type 1 in the Isolated Villous Trophoblast.. AIDS Res Hum Retroviruses.

[14] Weissman D, Poli G, Fauci AS (1994). Interleukin 10 blocks HIV replication in macrophages by inhibiting the autocrine loop of tumor necrosis factor alpha and interleukin 6 induction of virus.. AIDS Res Hum Retroviruses.

[15] McGowan I, Elliott J, Fuerst M, Taing P, Boscardin J (2004). Increased HIV-1 mucosal replication is associated with generalized mucosal cytokine activation.. J Acquir Immune Defic Syndr.

[16] Moussa M, Mognetti B, Dubanchet S, Menu E, Roques P (1999). Vertical transmission of HIV: parameters which might affect infection of placental trophoblasts by HIV-1: a review. Biomed Group on the Study of in Utero Transmission of HIV 1.. Am J Reprod Immunol.

[17] Patterson BK, Behbahani H, Kabat WJ, Sullivan Y, O'Gorman MR (2001). Leukemia inhibitory factor inhibits HIV-1 replication and is upregulated in placentae from nontransmitting women.. J Clin Invest.

[18] Menu E, Mognetti B, Moussa M, Nardese V, Tresoldi L (1997). Insights into the mechanisms of vertical transmission of HIV-1. BIOMED2 Working Group on the in utero transmission of HIV-1.. Early Pregnancy.

[19] Coulomb-L'Hermine A, Emilie D, Durand-Gasselin I, Galanaud P, Chaouat G (2000). SDF-1 production by placental cells: a potential mechanism of inhibition of mother-to-fetus HIV transmission.. AIDS Res Hum Retroviruses.

[20] Behbahani H, Popek E, Garcia P, Andersson J, Spetz AL (2000). Up-regulation of CCR5 expression in the placenta is associated with human immunodeficiency virus-1 vertical transmission.. Am J Pathol.

[21] Kfutwah AK, Mary JY, Nicola MA, Blaise-Boisseau S, Barré-Sinoussi F (2006). Tumour necrosis factor-alpha stimulates HIV-1 replication in single-cycle infection of human term placental villi fragments in a time, viral dose and envelope dependent manner.. Retrovirology.

[22] Foli A, Saville MW, May LT, Webb DS, Yarchoan R (1997). Effects of human immunodeficiency virus and colony-stimulating factors on the production of interleukin 6 and tumor necrosis factor alpha by monocyte/macrophages.. AIDS Res Hum Retroviruses.

[23] Kedzierska K, Crowe SM (2001). Cytokines and HIV-1: interactions and clinical implications.. Antivir Chem Chemother.

[24] Fried M, Duffy PE (1996). Adherence of Plasmodium falciparum to chondroitin sulfate A in the human placenta.. Science.

[25] Fried M, Muga RO, Misore AO, Duffy PE (1998). Malaria elicits type 1 cytokines in the human placenta: IFN-gamma and TNF-alpha associated with pregnancy outcomes.. J Immunol.

[26] O'Neil-Dunne I, Achur RN, Agbor-Enoh ST, Valiyaveettil M, Naik RS (2001). Gravidity-dependent production of antibodies that inhibit binding of Plasmodium falciparum-infected erythrocytes to placental chondroitin sulfate proteoglycan during pregnancy.. Infect Immun.

[27] Duffy PE, Fried M (2003). Antibodies that inhibit Plasmodium falciparum adhesion to chondroitin sulfate A are associated with increased birth weight and the gestational age of newborns.. Infect Immun.

[28] Salanti A, Dahlbäck M, Turner L, Nielsen MA, Barfod L (2004). Evidence for the involvement of VAR2CSA in pregnancy-associated malaria.. J Exp Med.

[29] Staalsoe T, Shulman CE, Bulmer JN, Kawuondo K, Marsh K (2004). Variant surface antigen-specific IgG and protection against clinical consequences of pregnancy-associated Plasmodium falciparum malaria.. Lancet.

[30] Moormann AM, Sullivan AD, Rochford RA, Chensue SW, Bock PJ (1999). Malaria and pregnancy: placental cytokine expression and its relationship to intrauterine growth retardation.. J Infect Dis.

[31] Fievet N, Moussa M, Tami G, Maubert B, Cot M (2001). Plasmodium falciparum induces a Th1/Th2 disequilibrium, favoring the Th1-type pathway, in the human placenta.. J Infect Dis.

[32] Suguitan AL, Cadigan TJ, Nguyen TA, Zhou A, Leke RJ (2003). Malaria-associated cytokine changes in the placenta of women with pre-term deliveries in Yaounde, Cameroon.. Am J Trop Med Hyg.

[33] Rogerson SJ, Brown HC, Pollina E, Abrams ET, Tadesse E (2003). Placental Tumor Necrosis Factor Alpha but Not Gamma Interferon Is Associated with Placental Malaria and Low Birth Weight in Malawian Women.. Infect Immun.

[34] Moore JM, Chaisavaneeyakorn S, Perkins DJ, Othoro C, Otieno J (2004). Hemozoin Differentially Regulates Proinflammatory Cytokine Production in Human Immunodeficiency Virus-Seropositive and -Seronegative Women with Placental Malaria.. Infect Immun.

[35] O'Brien ME, Kupka R, Msamanga GI, Saathoff E, Hunter DJ (2005). Anemia is an independent predictor of mortality and immunologic progression of disease among women with HIV in Tanzania.. J Acquir Immune Defic Syndr.

[36] Bodkin C, Klopper H, Langley G (2006). A comparison of HIV positive and negative pregnant women at a public sector hospital in South Africa.. J Clin Nurs.

[37] Biswas P, Mantelli B, Sica A, Malnati M, Panzeri C (2003). Expression of CD4 on human peripheral blood neutrophils.. Blood.

[38] Salmen S, Terán G, Borges L, Goncalves L, Albarrán B (2004). Increased Fas-mediated apoptosis in polymorphonuclear cells from HIV-infected patients.. Clin Exp Immunol.

[39] Shearer WT, Reuben J, Lee BN, Popek EJ, Lewis DE (1997). Role of placental cytokines and inflammation in vertical transmission of HIV infection.. Acta Paediatr Suppl.

[40] Faye A, Pornprasert S, Mary JY, Dolcini G, Derrien M (2007). Characterization of the main placental cytokine profiles from HIV-1-infected pregnant women treated with anti-retroviral drugs in France.. Clin Exp Immunol.

[41] Suguitan AL, Leke RG, Fouda G, Zhou A, Thuita L (2003). Changes in the Levels of Chemokines and Cytokines in the Placentas of Women with Plasmodium falciparum Malaria.. J Infect Dis.

[42] Chaisavaneeyakorn S, Moore JM, Mirel L, Othoro C, Otieno J (2003). Levels of macrophage inflammatory protein 1 alpha (MIP-1 alpha) and MIP-1 beta in intervillous blood plasma samples from women with placental malaria and human immunodeficiency virus infection.. Clin Diagn Lab Immunol.

[43] Israel N, Hazan U, Alcami J, Munier A, Arenzana-Seisdedos F (1989). Tumor necrosis factor stimulates transcription of HIV-1 in human T lymphocytes, independently and synergistically with mitogens.. J Immunol.

[44] Vyakarnam A, McKeating J, Meager A, Beverley PC (1990). Tumour necrosis factors (alpha, beta) induced by HIV-1 in peripheral blood mononuclear cells potentiate virus replication.. Aids.

[45] Lane BR, Markovitz DM, Woodford NL, Rochford R, Strieter RM (1999). TNF-alpha inhibits HIV-1 replication in peripheral blood monocytes and alveolar macrophages by inducing the production of RANTES and decreasing C-C chemokine receptor 5 (CCR5) expression.. J Immunol.

[46] Velez DR, Fortunato SJ, Morgan N, Edwards TL, Lombardi SJ (2008). Patterns of cytokine profiles differ with pregnancy outcome and ethnicity.. Hum Reprod.

[47] Ng SC, Gilman-Sachs A, Thaker P, Beaman KD, Beer AE (2002). Expression of intracellular Th1 and Th2 cytokines in women with recurrent spontaneous abortion, implantation failures after IVF/ET or normal pregnancy.. Am J Reprod Immunol.

[48] Tako EA, Zhou A, Lohoue J, Leke R, Taylor DW (2005). Risk factors for placental malaria and its effect on pregnancy outcome in Yaounde, Cameroon.. Am J Trop Med Hyg.

[49] Ayouba A, Nerrienet E, Menu E, Lobe MM, Thonnon J (2003). Mother-to-child transmission of human immunodeficiency virus type 1 in relation to the season in Yaounde, Cameroon.. Am J Trop Med Hyg.

[50] Skinner-Adams TS, McCarthy JS, Gardiner DL, Andrews KT (2008). HIV and malaria co-infection: interactions and consequences of chemotherapy.. Trends Parasitol.

[51] Pornprasert S, Mary JY, Faye A, Leechanachai P, Limtrakul A (2009). Higher placental anti-inflammatory IL-10 cytokine expression in HIV-1 infected women receiving longer zidovudine prophylaxis associated with nevirapine.. Curr HIV Res.

[52] Ayouba A, Tene G, Cunin P, Foupouapouognigni Y, Menu E (2003). Low rate of mother-to-child transmission of HIV-1 after nevirapine intervention in a pilot public health program in Yaounde, Cameroon.. J Acquir Immune Defic Syndr.

[53] Taupin JL, Gualde N, Moreau JF (1997). A monoclonal antibody based ELISA for quantitation of human leukaemia inhibitory factor.. Cytokine.

[54] Pornprasert S, Faye A, Mary JY, Dolcini G, Leechanachai P (2006). Down Modulation of TNF-a mRNA Placental Expression by AZT Used for the Prevention of HIV-1 Mother-to-Child Transmission.. Placenta.

