# Supplementary figures and images for: Plasmodium falciparum Infection Significantly Impairs Placental Cytokine Profile in HIV Infected Cameroonian Women
Source: PLoS One. 2009 Dec 2;4(12):e8114. doi: 10.1371/journal.pone.0008114 (PMC2780732; doi:10.1371/journal.pone.0008114)

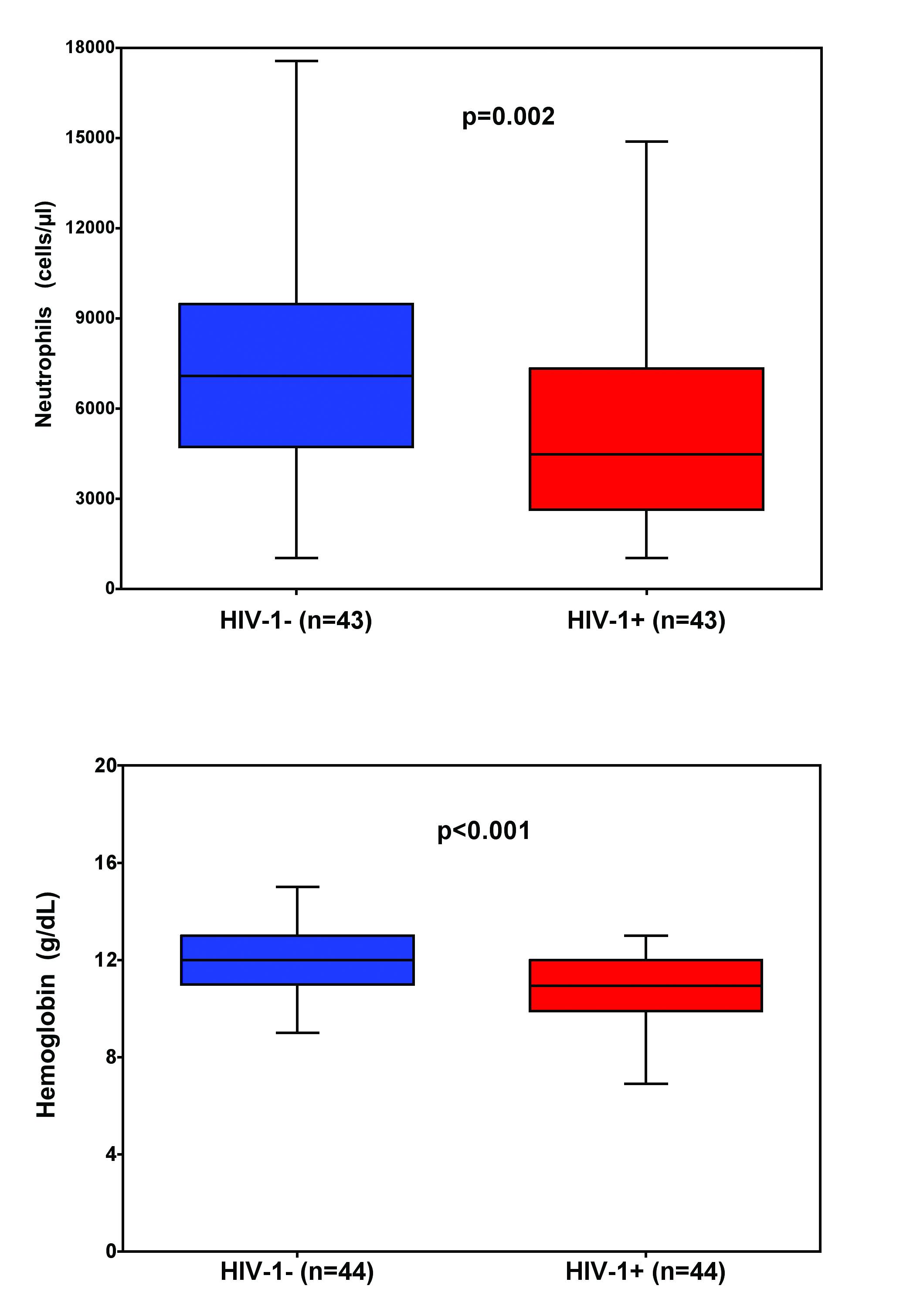

Supplement: Figure S1 — Box-plot of hemoglobin (bottom panel) and neutrophil (upper panel) distributions in the blood of HIV-1 negative and positive pregnant women. Hemoglobin and neutrophil levels were quantified in blood samples of pregnant women by an automated counter. Distributions are represented per group as median, inter-quartile range, 95% confidence interval and values outside this interval. Comparisons between the 2 HIV-1 groups were performed through Mann Whitney test. (1.16 MB TIF) [file pone.0008114.s001.tif]
